# HotSpotter: efficient visualization of driver mutations

**DOI:** 10.1186/1471-2164-15-1044

**Published:** 2014-12-01

**Authors:** Jason Roszik, Scott E Woodman

**Affiliations:** Department of Melanoma Medical Oncology, The University of Texas MD Anderson Cancer Center, 7455 Fannin St, Houston, TX 77054 USA; Department of Systems Biology, The University of Texas MD Anderson Cancer Center, 7455 Fannin St, Houston, TX 77054 USA

**Keywords:** Cancer, Driver mutation, Hotspots, Visualization

## Abstract

**Background:**

Driver mutations are positively selected during the evolution of cancers. The relative frequency of a particular mutation within a gene is typically used as a criterion for identifying a driver mutation. However, driver mutations may occur with relative infrequency at a particular site, but cluster within a region of the gene. When analyzing across different cancers, particular mutation sites or mutations within a particular region of the gene may be of relatively low frequency in some cancers, but still provide selective growth advantage.

**Results:**

This paper presents a method that allows rapid and easy visualization of mutation data sets and identification of potential gene mutation hotspot sites and/or regions. As an example, we identified hotspot regions in the NFE2L2 gene that are potentially functionally relevant in endometrial cancer, but would be missed using other analyses.

**Conclusions:**

HotSpotter is a quick, easy-to-use visualization tool that delivers gene identities with associated mutation locations and frequencies overlaid upon a large cancer mutation reference set. This allows the user to identify potential driver mutations that are less frequent in a cancer or are localized in a hotspot region of relatively infrequent mutations.

**Electronic supplementary material:**

The online version of this article (doi:10.1186/1471-2164-15-1044) contains supplementary material, which is available to authorized users.

## Background

**Figure 1:**
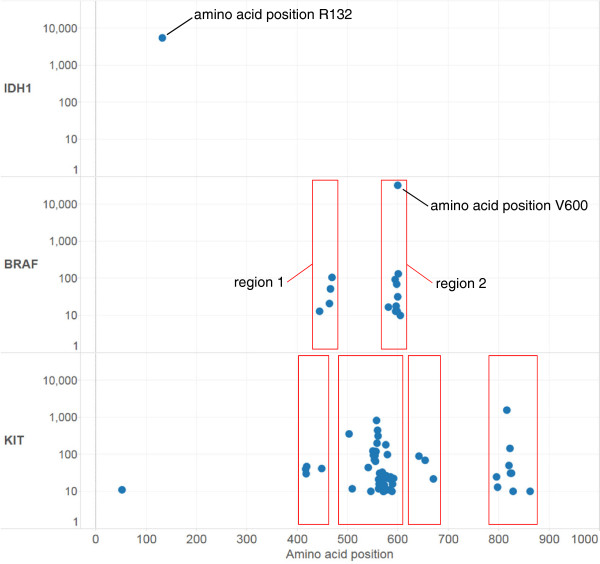
**Driver mutation amino acid sites and regions.** The COSMIC dataset was used to identify the most frequent mutations for well-characterized driver mutation sites/regions. The y-axis demarcates the names of genes and frequency of mutations within each gene. The x-axis demarcates the amino acid position within the protein product for each mutation. The vertical position of the blue dots represents the frequency of an amino acid alteration at a specific site. For illustrative purposes a few well-characterized mutant proteins are displayed, and a threshold of at least 10 samples with a specific amino acid aberration was employed. For the IDH1 protein, the R132 amino acid site is clearly the most aberrant (n = 5,557 entries) to the exclusion of any other site. The BRAF protein displays both a dominant site at V600 (n = 32,371 entries) within one of two relatively high frequency mutation regions. Finally, the KIT protein demonstrates regions with relatively high frequencies of amino acid alterations.

Driver mutations provide a growth advantage for tumor cells and have been positively selected during the evolution of a cancer [1]. When exploring the genetic underpinnings or searching for possible therapeutic targets in cancer, it is very important to be able to identify potential driver mutations. Driver mutations are often distinguished from passenger mutations by determining the difference in frequency at a particular location within a gene that results in a functional alteration of the protein product [2]. Driver mutations may be exhibited as alterations (missense, deletion, insertion, termination, etc.) that occur at a higher frequency within a particular region within a protein and/or as a high frequency alteration starting at a specific amino acid site. Figure 1 demonstrates driver mutations that localize at specific amino acid regions or sites.

However, what is considered a significant mutation frequency varies among approaches. Some approaches use frequency within the cancer of interest, while others use the frequency across cancers [2]. The difference in these approaches is highlighted by the recent analysis of the well-established activating BRAF hotspot missense mutations resulting in alterations at the V600 amino acid position in multiple tumor types [3, 4]. The BRAF V600 hotspot mutation occurs with a frequency of ~45% in melanoma and papillary thyroid cancer, and about ~10% in colorectal cancer, and the same mutation is typically observed in 0-4% of most other cancers. The use of specific small molecule inhibitors of mutant BRAF in patients with tumors harboring a BRAF V600 mutation has demonstrated that neither within, nor across, cancer frequency is sufficient to determine the functional significance of inhibiting “driver” mutations [5].

The HotSpotter method for identifying potential mutation hotspot sites and/or regions is agnostic as to whether mutation frequencies are within or across cancer types. The method is easily adaptable to any reference database of somatic mutations. For this application, version 66 of the Catalogue of Somatic Mutations in Cancer (COSMIC) [6] containing 1,524,610 entries was chosen.

Resources such as the cBio Portal [7] or the UCSC Cancer Genomics Browser [8] are excellent for reviewing publicly available data sets such as the Cancer Genome Atlas [9]. However, an easy-to-use application for determining potential mutation hotspot sites/regions from one’s own or publicly available mutation data sets would be of great value to both those with experience and those less experienced in exploring genomics results. This report summarizes the development and testing of such an application, which we named HotSpotter. HotSpotter allows users to:

1. easily visualize potential driver mutations, especially if the specific mutation is less frequent in the sample set being analyzed,
2. spot potential driver mutations that localize across a region of a gene,
3. easily filter samples with the threshold frequency desired,
4. analyze tumor mutation data without absolute dependence on a normal control, and
5. easily add one’s own or publicly available mutation data to enrich either the test or reference database.

## Results and discussion

To illustrate the strength of the HotSpotter method, we used mutation calls derived from exome sequencing data of 248 tumors previously published by the TCGA uterine corpus endometrial cancer (UCEC) work group [10]. To eliminate self-referential observations, a COSMIC data set devoid of the TCGA UCEC endometrial cancer samples was employed as the reference mutation data set. HotSpotter displayed the frequency of every mutation call in the TCGA UCEC dataset relative to that in the COSMIC dataset. Manual visualization of the results quickly identifies the potential mutation hotspot sites and/or regions within the TCGA UCEC samples; these potential hotspots appear as large orange dots. These dots are intentionally larger than the smaller blue dots representing all the mutation calls for the particular gene in the COSMIC database, on which they are overlaid. This overlaying of the two plots allows effective visualization of the potential driver mutations.

**Figure 2:**
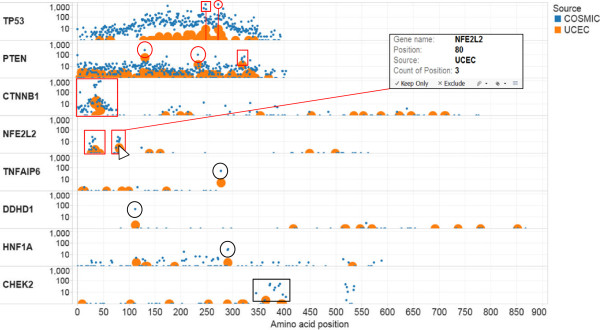
**HotSpotter identification of potential mutation hotspot sites/regions in the TCGA UCEC data set.** The y-axis demarcates the names of genes and frequency of mutations within each gene. The x-axis demarcates the amino acid position within the protein product for each mutation. Orange dots (intentionally large for quick visualization) and their vertical position represent the frequency of mutation at a specific site in the UCEC test set. Blue dots (intentionally smaller) and their vertical position represent the frequency of mutations at specific sites in the COSMIC dataset. For non-substitution mutations, the first amino acid at which the alteration occurs is used as the “position”.

Figure 2 shows how HotSpotter illustrated potential mutation hotspot amino acid sites and/or regions derived from the TCGA UCEC samples (The Tableau interface can be downloaded at the following address: http://public.tableausoftware.com/views/HotSpotter-TCGA_UCEC_selected/Selectedgenes). The y-axis demarcates the names of genes and the frequency of specific amino acid alterations arising from specific mutations (hereafter, termed ‘mutations’) within each gene. The x-axis demarcates the amino acid position within the inferred protein product for each mutation.

In the first row of Figure 2, the frequency of individual TP53 mutations within the TCGA UCEC sample set are shown (orange dots). Clearly, one of the TP53 mutations within the UCEC sample set falls within a region with a high frequency of mutations within the COSMIC database (three distinct mutations with approximately 500, 600 and 1300 entries, respectively, demarcated by the red rectangle). The other highest frequency TP53 mutation site within the TCGA UCEC dataset is also one of the top TP53 mutation sites within COSMIC (Figure 2, row one, red circle, 1225 entries).

Mutations of the PTEN gene were more widely dispersed throughout the gene than the TP53 mutations in both the TCGA UCEC samples and the COSMIC samples. Notably, HotSpotter readily illustrates the two highest frequency mutation sites within the TCGA UCEC sample set to be among the highest frequency mutation sites in COSMIC (Figure 2, row two, red circles). The other high frequency PTEN mutation in the TCGA UCEC sample set appears to locate to a highly mutated region in PTEN, as demonstrated by the presence of four closely grouped mutations (Figure 2, row two, red rectangle).

HotSpotter shows mutations within the CTNNB1 and NFE2L2 genes in the TCGA UCEC samples to have a clear overlap with highly mutated regions within these genes in COSMIC (Figure 2, rows 3–4, red boxes). NFE2L2 mutations within the TCGA UCEC dataset would not be considered “recurrent mutations”, as they are present only between one to three times at any one site. However, when superimposed on the visualization of NFE2L2 mutations in COSMIC, there is clearly overlap in amino acid regions 23–48 and 71–86. Scrolling over any specific mutation in HotSpotter (see Figure 2 inset) reveals additional information. In this particular case, the pointer arrow overlies the NFE2L2 mutation with the highest frequency (highest orange dot) in the TCGA UCEC samples. The inset box reveals the gene’s name, amino acid position, source, and count of position (i.e., the number of samples with mutations at that specific position). With this method, any one mutation or set of mutations can be selected and the extensive underlying data can be explored further by clicking on the data set icon (Additional file 1: Figure S1A and B). The underlying data shows that each of the NFE2L2 mutation sites in endometrial cancer is also a mutation site in a variety of other cancers, primarily in squamous cell carcinomas. Interestingly, the same two regions were identified by Sasaki et al. [11], who showed that mutations in those regions were associated with significantly worse prognosis in lung squamous cell carcinoma. Of interest, these NFE2L2 mutation regions observed by the HotSpotter method were not noted in the recently published TCGA report on the genomic characterization of UCEC [10]. Similarly, low-frequency mutations in DDHD1, HNF1A and CHEK2 within endometrial cancer samples were shown to be highly mutated sites/regions in multiple cancers in the COSMIC database (Figure 2, rows 5–8, black circles/box).

## Conclusions

HotSpotter offers a rapid, easy-to-use method for analyzing one’s own and/or publicly available mutation data sets (as shown with the TCGA UCEC data set). This approach is, of course, limited by the particular features of the reference mutation data set employed, e.g. inclusion of cell lines and tissues, over- and under- representation of cancer types, the lack of a definitive sample denominator for many mutations, and redundancy of samples, etc. However, a strength of HotSpotter is its flexibility; it is agnostic to the particular methods that precede its use (e.g., mutation calling algorithms, false discovery rates, etc.). Essentially the same approach taken here can be used with most reference databases, other than COSMIC. HotSpotter provides a very quick tool delivering gene identities with associated mutation locations and frequencies overlaid upon a cancer mutation reference set (>1.5 million mutation calls in the example given). The viewer can rapidly see specific gene mutation sites and/or region patterns, and ready access to structured underlying data is also provided, upon which further analyses can be performed.

## Methods

The Tableau Desktop business intelligence software [12] was employed to create an interface that displays the frequency/location of particular amino acid alterations derived from gene mutations identified within the TCGA UCEC sample set overlaid upon the frequency/location of particular amino acid alterations derived from mutations within the same gene within the COSMIC database version 66 [6].

The following process was used to prepare a modified COSMIC reference dataset. The amino acid mutation position data was extracted from the “Mutation AA” column of the COSMIC data set for every mutation and appended to the COSMIC data table as a new column entitled “Position”. Another new column which must be appended to the COSMIC data table is the column entitled “Source”. The term ‘COSMIC’ was used for each cell of the Source column for this analysis. After performing these steps, the user can replace the COSMIC dataset with any dataset of their choosing.

For the sample mutation dataset (TCGA UCEC), the same steps used for the modified COSMIC reference dataset must be performed. In addition, the rows of the sample mutation dataset to be analyzed must be appended to the modified reference dataset. Mandatory columns for the reference and the sample data sets are the following: “Gene name”, “Mutation AA”, “Position”, and “Source”. The data table can contain additional columns, and the position of the columns has no effect on the interface. The only requirement is to have a table that contains the reference/sample mutations, and the table must have the four columns as described above. To be able to differentiate between the reference and the sample dataset, distinct names in the “Source” column are necessary. The modified dataset can be easily updated in Tableau using the “Edit connection” (Data menu - > [Reference dataset] - > Edit Connection) menu item.

## Electronic supplementary material

Additional file 1: Figure S1: A) Highlighting mutation calls that arise within two specific regions of the NFE2L2 gene from the TCGA UCEC and COSMIC datasets. The underlying data for the selected NFE2L2 gene mutation, and the tumor samples from which they were derived, can be viewed by selecting the data set icon located on the bottom right aspect of the inset when the mouse pointer overlays any highlighted sample(s). B) Selected data types within the underlying data from the TCGA UCEC and COSMIC datasets that correspond to the highlighted NFE2L2 mutations in Figure S1A. The data has been ranked first by source, then by position at which the alteration is localized within the protein. For non-substitution mutations, the amino at which the alteration occurs is noted as the “position”. (PPTX 833 KB)

## Abbreviations

COSMIC: Catalogue of Somatic Mutations in Cancer
TCGA: The Cancer Genome Atlas
UCEC: Uterine corpus endometrial carcinoma.
