# Supplementary material for: HotSpotter: efficient visualization of driver mutations
Source: BMC Genomics. 2014 Dec 1;15(1):1044. doi: 10.1186/1471-2164-15-1044 (PMC4265503; doi:10.1186/1471-2164-15-1044)
Supplement: Supplementary file 1 — Additional file 1: Figure S1: A) Highlighting mutation calls that arise within two specific regions of the NFE2L2 gene from the TCGA UCEC and COSMIC datasets. The underlying data for the selected NFE2L2 gene mutation, and the tumor samples from which they were derived, can be viewed by selecting the data set icon located on the bottom right aspect of the inset when the mouse pointer overlays any highlighted sample(s). B) Selected data types within the underlying data from the TCGA UCEC and COSMIC datasets that correspond to the highlighted NFE2L2 mutations in Figure S1A. The data has been ranked first by source, then by position at which the alteration is localized within the protein. For non-substitution mutations, the amino at which the alteration occurs is noted as the “position”. (PPTX 833 KB) [file 12864_2014_6748_MOESM1_ESM.pptx]

## Slide 1
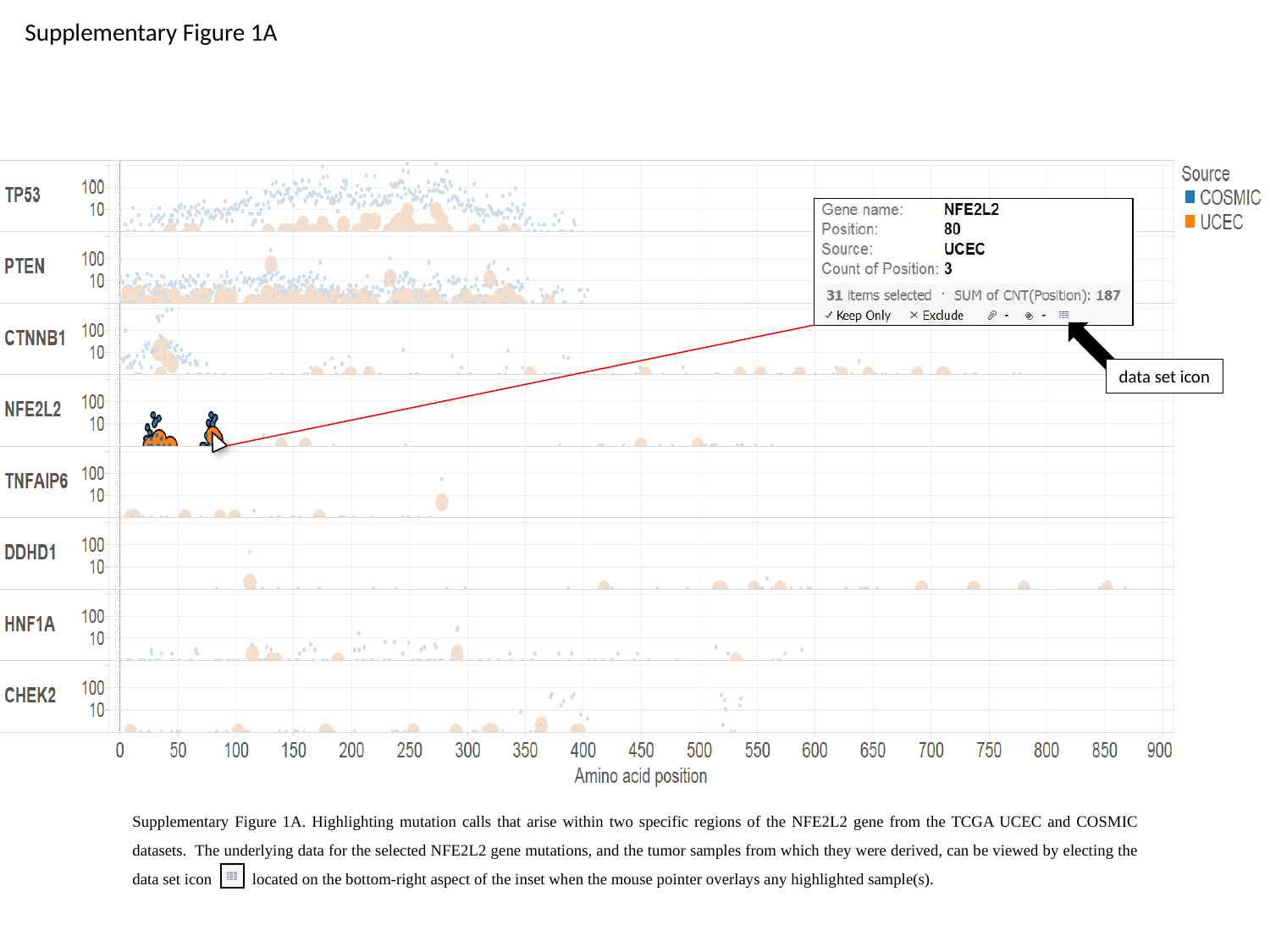

Supplementary Figure 1A
Supplementary Figure 1A. Highlighting mutation calls that arise within two specific regions of the NFE2L2 gene from the TCGA UCEC and COSMIC datasets. The underlying data for the selected NFE2L2 gene mutations, and the tumor samples from which they were derived, can be viewed by electing the data set icon located on the bottom-right aspect of the inset when the mouse pointer overlays any highlighted sample(s).
data set icon

## Slide 2
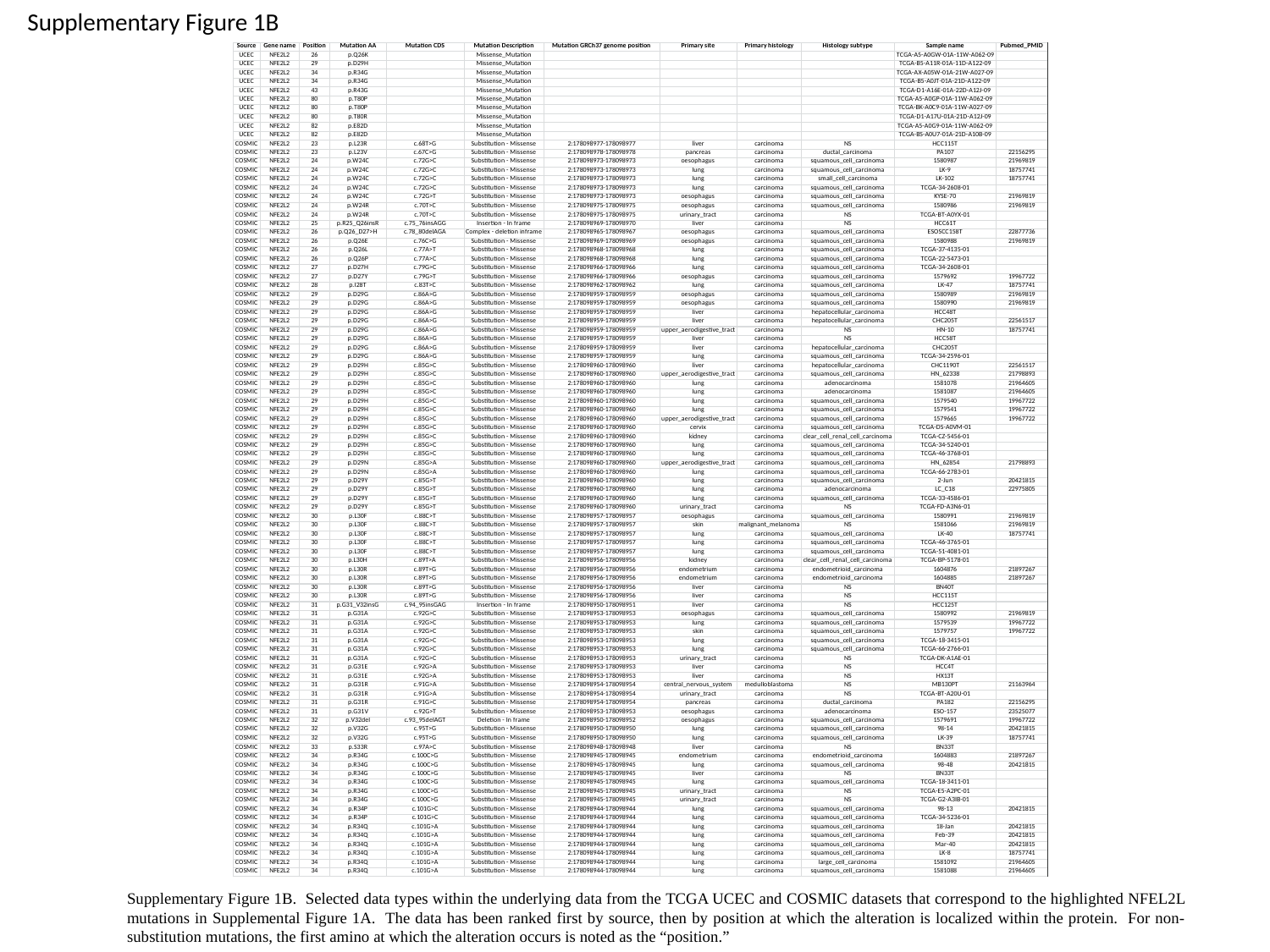

Supplementary Figure 1B
Supplementary Figure 1B. Selected data types within the underlying data from the TCGA UCEC and COSMIC datasets that correspond to the highlighted NFEL2L mutations in Supplemental Figure 1A. The data has been ranked first by source, then by position at which the alteration is localized within the protein. For non-substitution mutations, the first amino at which the alteration occurs is noted as the “position.”

## Slide 3
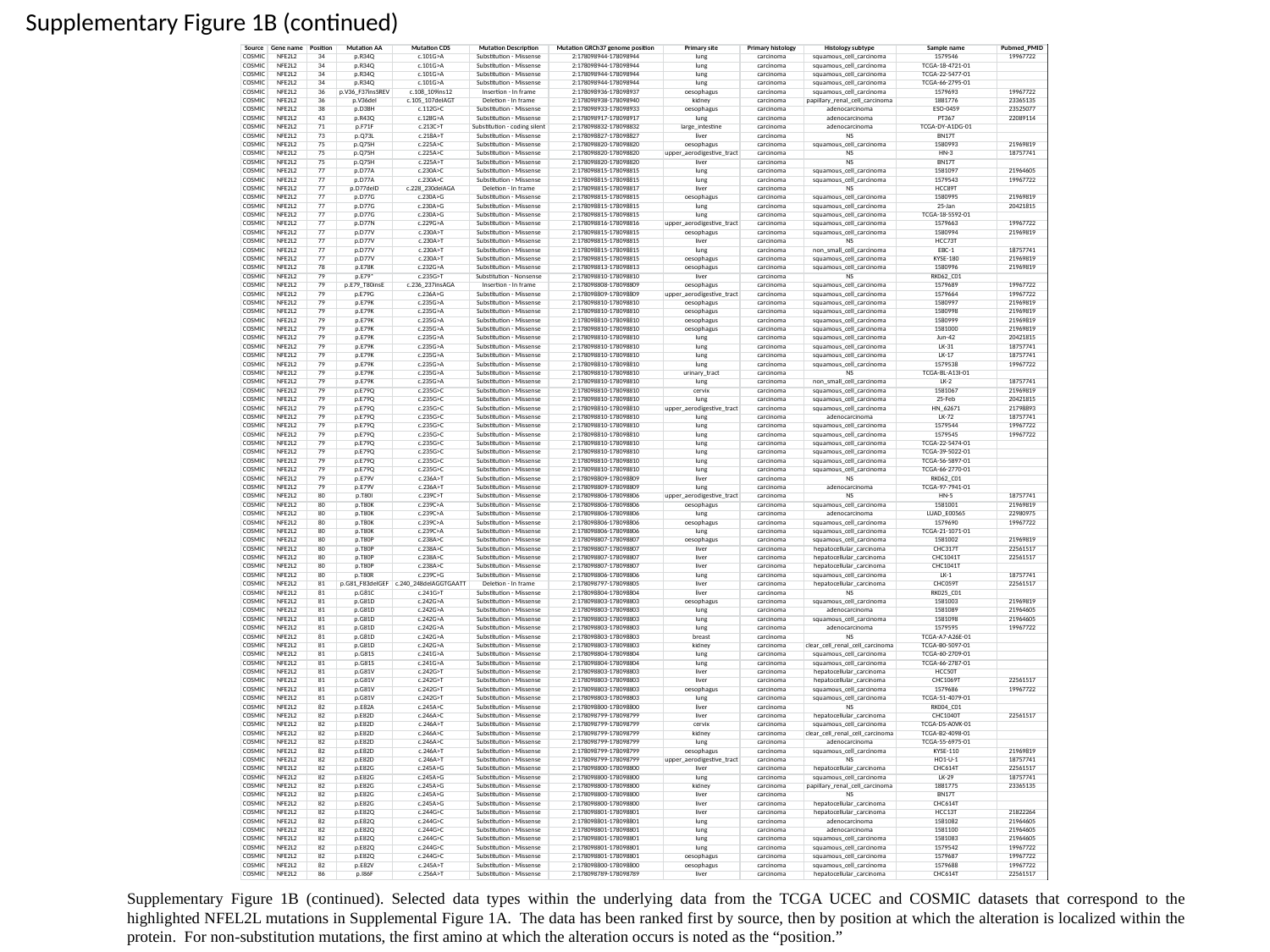

Supplementary Figure 1B (continued)
Supplementary Figure 1B (continued). Selected data types within the underlying data from the TCGA UCEC and COSMIC datasets that correspond to the highlighted NFEL2L mutations in Supplemental Figure 1A. The data has been ranked first by source, then by position at which the alteration is localized within the protein. For non-substitution mutations, the first amino at which the alteration occurs is noted as the “position.”
